# Phosphodiesterase 10A levels are related to striatal function in schizophrenia: a combined positron emission tomography and functional magnetic resonance imaging study

**DOI:** 10.1007/s00406-019-01021-0

**Published:** 2019-05-22

**Authors:** Jonas Persson, K. Szalisznyó, G. Antoni, A. Wall, D. Fällmar, H. Zora, R. Bodén

**Affiliations:** 1 Department of Neuroscience, Psychiatry, Uppsala University, Uppsala, Sweden; 2 Department of Medicinal Chemistry, Uppsala University, Uppsala, Sweden; 3 PET-Centre, Uppsala University Hospital, Uppsala, Sweden; 4 Department of Surgical Sciences, Radiology, Uppsala University, Uppsala, Sweden; 5 Department of Linguistics, Stockholm University, Stockholm, Sweden; 6 Department of Surgical Sciences, Nuclear medicine and PET, Uppsala University, Uppsala, Sweden

**Keywords:** Phosphodiesterase 10A, Schizophrenia, Striatum, Dopamine, Resting state

## Abstract

Pharmacological inhibition of phosphodiesterase 10A (PDE10A) is being investigated as a treatment option in schizophrenia. PDE10A acts postsynaptically on striatal dopamine signaling by regulating neuronal excitability through its inhibition of cyclic adenosine monophosphate (cAMP), and we recently found it to be reduced in schizophrenia compared to controls. Here, this finding of reduced PDE10A in schizophrenia was followed up in the same sample to investigate the effect of reduced striatal PDE10A on the neural and behavioral function of striatal and downstream basal ganglia regions. A positron emission tomography (PET) scan with the PDE10A ligand [^11^C]Lu AE92686 was performed, followed by a 6 min resting-state magnetic resonance imaging (MRI) scan in ten patients with schizophrenia. To assess the relationship between striatal function and neurophysiological and behavioral functioning, salience processing was assessed using a mismatch negativity paradigm, an auditory event-related electroencephalographic measure, episodic memory was assessed using the Rey auditory verbal learning test (RAVLT) and executive functioning using trail-making test B. Reduced striatal PDE10A was associated with increased amplitude of low-frequency fluctuations (ALFF) within the putamen and substantia nigra, respectively. Higher ALFF in the substantia nigra, in turn, was associated with lower episodic memory performance. The findings are in line with a role for PDE10A in striatal functioning, and suggest that reduced striatal PDE10A may contribute to cognitive symptoms in schizophrenia.

## Introduction

Established antipsychotic treatments in schizophrenia act mainly by blocking dopamine D_2_ receptors in the striatum and are generally effective in reducing positive symptoms. However, negative and cognitive symptoms are often refractory to antipsychotic treatment and have a deleterious impact on quality of life and social functioning [1, 2]. Consequently, there is a great need for new treatments to target this broad range of symptoms.

### Phosphodiesterase 10A

One possible treatment option is the pharmacological inhibition of phosphodiesterase 10A (PDE10A) [3–5]. PDE10A is an enzyme that is highly concentrated and localized in striatal medium spiny neurons (MSN) across mammalian species [6]. In the striatum, PDE10A regulates the excitability of MSNs mainly through the inactivation of the second messengers cyclic adenosine monophosphate (cAMP) and cyclic guanosine monophosphate (cGMP) [5, 7], thereby acting postsynaptically on dopamine signaling. Normally, D_1_ receptor activation stimulates cAMP production, while D_2_ receptor activation instead has the opposite effect. Given the postsynaptic role of PDE10A, its inactivation enhances the effect of dopamine D_1_ receptor activation in the striatonigral, direct, pathway. Simultaneously, PDE10A inhibition counteracts the normally inhibitory effect of D_2_ receptor signaling in the striatopallidal, indirect, pathway [8], making it an attractive target for antipsychotic drugs.

Indeed, PDE10A inhibitors have shown promising results across a range of symptoms in preclinical schizophrenia models [3–5]. However, preliminary trials in humans have been less encouraging [9, 10]. If the inhibition of PDE10A is indeed a viable treatment option, an increase in striatal PDE10A levels in schizophrenia would be expected. However, we recently found that striatal PDE10A was reduced in patients with schizophrenia compared to controls [11]. Understanding whether and how this relates to changes in brain function in the striatum and its associated symptoms would help to clarify the role of PDE10A in schizophrenia.

### Striatal function in schizophrenia

The observation that virtually all antipsychotic medications are D_2_-receptor antagonists suggested involvement of the striatum in schizophrenia, and later positron emission tomography (PET) studies have confirmed elevated synaptic dopamine in the associative striatum [12–14].

Similarly, striatal functions have been studied in schizophrenia using resting-state functional magnetic resonance imaging (fMRI). Both fMRI-derived measures of functional connectivity and regional activity have been considered, the latter measured as the amplitude of low-frequency fluctuations (ALFF) [15, 16]. Increased ALFF in the bilateral putamen has been repeatedly observed in treatment-naive patients [17–19], as well as in medicated patients [20] compared to healthy controls, which could indicate that current treatments do not normalize this hyperactivity, in line with a limited effect in cognitive impairment. Further, a recent meta-analysis revealed altered striatal resting-state connectivity in schizophrenia, compared to controls, with emotion processing and salience network regions [21]. This is in line with striatal dopamine signaling being linked to salience network connectivity in healthy participants [22]. This may reflect aberrant salience processing in patients with schizophrenia. A well-replicated finding in schizophrenia is a reduction in the mismatch negativity (MMN) response, an event-related potential (ERP) reflecting processing of salient stimuli [23]. Altered salience network activity in schizophrenia has been observed during an MMN paradigm [24]. Striatal dopamine signaling is also associated with executive and memory functions, including working and episodic memory [25–28]. While episodic memory is primarily associated with the medial temporal lobe, performance was found to be strongly associated with putamen volume in schizophrenia [29]. In healthy individuals, episodic memory performance has been linked to striatal D_2_-receptor binding [27], which modulates hippocampal–striatal connectivity during episodic memory [30], and to activation of the substantia nigra [31]. Similarly, episodic memory impairments are seen in Parkinson’s disease as well, possibly reflecting impaired retrieval [32]. Performance on the trail-making test B (TMT-B) in turn, tapping into executive functioning, has been related to striatal D2/3-receptor availability, as measured with [^18^F]fallypride, in patients with schizophrenia [28]. Thus, altered striatal functioning may be related to both aberrant salience processing and cognitive deficits in schizophrenia.

In summary, there is ample evidence for aberrant striatal functioning in schizophrenia, but while increased activity in the striatum in rodents following PDE10A inhibition has been observed [33], the relationship between striatal function and PDE10A in schizophrenia is still unknown.

The aim of this study was to investigate whether an earlier reported reduction in striatal PDE10A levels [11] relates to striatal function in schizophrenia. The relationship between PDE10A availability and ALFF within the striatum and its basal ganglia output regions was assessed. For any region showing such a relationship, ALFF was in turn correlated to MMN as a measure of salience processing, as well as performance on cognitive measures associated with striatal functioning. Cognitive measures included verbal episodic memory, measured with the Rey Auditory Verbal Learning Test (RAVLT) [34] and one aspect of executive functioning, measured with TMT-B.

## Methods

### Participants and clinical data

The patients were the same as described in a prior publication [11], consisting of ten males between 18 and 45 years old, with a diagnosis of schizophrenia since at least 2 years prior to inclusion. One patient was treated with quetiapine, two with olanzapine, and among the remaining seven patients who was treated with clozapine, one patient had concomitant treatment with perphenazine, three with aripiprazole, one with quetiapine, and one with risperidone. Antipsychotic dosages were determined by reviewing prescription data in the medical records and the ingested doses were confirmed through patient interviews and monitoring of antipsychotic drug levels in blood. The dosages were converted to olanzapine equivalents to enable comparison [35]. The patients were asked to refrain from alcohol for 48 h prior to positron emission tomography (PET) scanning.

### Positron emission tomography

The PET data are the same as reported in a previous study, where the PET scanning and preprocessing procedures are described in more detail [11]. Briefly, an intravenous bolus injection of the PET tracer [^11^C]Lu AE92686, a PDE10A antagonist, was administered and data were collected during a 90 min dynamic scanning session. Reconstructed images were realigned to correct for head movements and co-registered to a T1-weighted anatomical image where a cerebellum volume of interest was defined as a reference region to compute parametric images, using a previously validated simplified reference tissue model [36].

### Magnetic resonance imaging

A 6-min resting-state fMRI scanning session was performed, where the patients were asked to keep their eyes closed and not think of anything in particular. MRI scans were made directly following PET scanning, in the afternoon.

MRI was performed on a 3 Tesla scanner (Philips Achieva dStream, Philips Medical Systems, Best, Netherlands), with a 32-channel head coil. Resting-state functional imaging was performed using single-shot gradient echo-planar imaging with interleaved acquisition, 32 slices with a thickness of 3 mm, TR/TE=2000/30 ms, field of view (FOV) = 192×192 mm^2^, voxel size = 27 mm^3^ isotropic voxels. T1-weighted structural images were acquired using a 3D turbo field echo sequence (repetition time = 8.2 ms; echo time = 3.8 ms; flip angle = 8°; field of view = 256 × 256 mm^2^; voxel size = 1 mm^3^ isotropic voxels; 220 slices, axial acquisition). Preprocessing of functional images was performed using DPARSF 4.3 Advanced edition [37] in Matlab R2017b. After the ten first time points were discarded, resting-state images were time-slice corrected and realigned to the first volume to correct for head motion. No participants exhibited head movements > 2.0 mm translation or 2° rotation. Nuisance regressors included head movements, using a 24-parameter model [38] as well as signal from white matter and cerebrospinal fluid. After co-registration to the structural image, functional images were normalized to MNI space through DARTEL and spatially smoothed with a Gaussian kernel of 4×4×4 mm^3^ FWHM.

ALFF was defined as the power across the frequency band 0.01–0.1 Hz based on the Fourier transform of the BOLD signal as described in Zang et al. 2007 [15], after subtracting the global signal. ALFF values were *z*-transformed by subtracting the whole-brain mean and dividing by the standard deviation.

### Electrophysiological measures

Behavioral and electrophysiological assessments were performed in the morning of the same day as the MRI and PET scanning sessions. The MMN paradigm was based on a previous study [39] and consisted of 1200 tones with a pitch of 1000 Hz, of which 1020 standard tones (85%) had a duration of 25 ms and 180 (15%) deviant tones had a duration of 50 ms. The interstimulus interval was 300 ms. EEG was monitored using Nexus-10 MKII and the Biotrace+ software from Mindmedia, with a single electrode at Fz according to the 10–20 positioning system. The reference electrode was placed on the nose and an electrode on the forehead was used for grounding. Calculation of the MMN response was done in Matlab R2017b. Data were bandpass filtered (1–20 Hz) using a Butterworth filter with order 4. Each epoch was defined as − 50 ms to 500 ms relative to stimulus onset. After subtracting the mean baseline, epochs were averaged for deviants and standards separately and deviant-minus-standard subtractions were carried out. MMN was defined as the most negative peak in the difference ERP within 80 and 130 ms post-stimulus onset.

### Behavioral measures

Here, a shortened version of the RAVLT was used [34]. In brief, a list of 15 words was read aloud to the patient at a constant rate, whereupon the patient was asked to immediately recall as many words as possible. After three repetitions, unrelated cognitive tasks were administered for 30 min after which the patient was asked to freely recall as many words as possible from the original list. The outcome measure was the number of correctly recollected words after the 30 min retention interval. TMT-A and -B [40] were administered here in a computerized version using Inquisit by Millisecond and a computer mouse. In TMT-A, the patient was asked to connect numbered dots sequentially by drawing a line, as quickly as possible. In TMT-B, the patient had to alternate between consecutive numbers and letters. While both TMT-A and TMT-B rely on motor functioning and visual scanning speed, especially TMT-B, which is considered here, is dependent on executive functioning [41].

### Analyses

PET non-displaceable binding potential (BP_ND_) volumes were co-registered to the structural T1-weighted images and normalized using the DARTEL parameters. Since significant BP_ND_ was observed exclusively in the striatum, only striatal PDE10A is considered here. Mean BP_ND_ was extracted from normalized volumes for each patient within a striatum region of interest (ROI) based on the CIC atlas [42], referred to as PDE10A level below. For defining regional ALFF values, basal ganglia ROIs were derived from the same atlas and were identical to those reported in a previous publication [11]. These included striatal regions (caudate nucleus, putamen and nucleus accumbens) as well as substantia nigra and globus pallidus from which mean ALFF values were extracted for each patient. Spearman correlations were calculated between regional ALFF and striatal BP_ND_, as well as between behavioral measures and ALFF within any region showing a significant relationship to striatal PDE10A. Correlations with a corresponding *p* value <0.05 were considered significant.

## Results

**Fig. 1:**
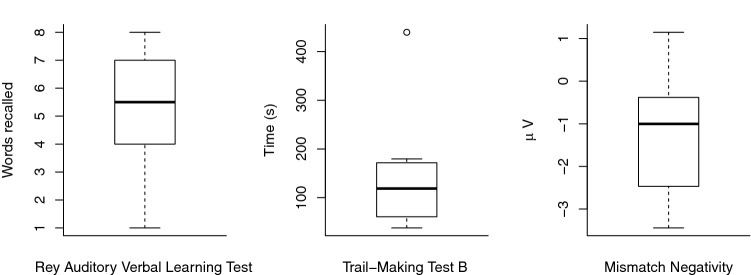
Box plots illustrating the performance on the behavioral and electrophysiological measures. From left to right: Rey Auditory Verbal Learning Test (RAVLT), trail-making test B (TMT-B), and mismatch negativity (MMN)

Behavioral and physiological measures are illustrated in Fig. 1. Median performance on RAVLT was 5.5 (IQR 2.75) correctly recalled words. On TMT-B, median time to completion was 118.86 (IQR 101.89) seconds. The median MMN amplitude was − 1.002 (2.002) µV.

**Fig. 2:**
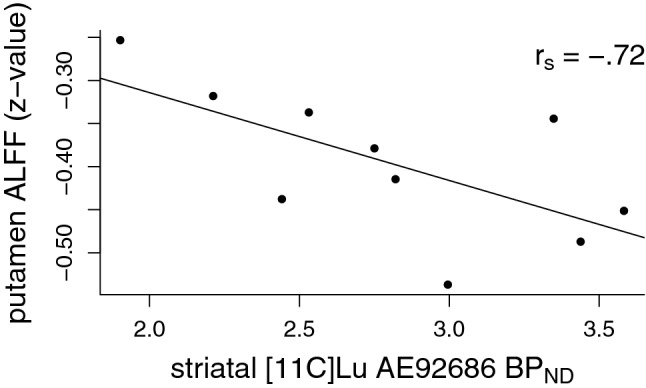
Scatter plot illustrating the correlation between mean striatal non-displaceable binding potential (BP_ND_) and mean putamen amplitude of low-frequency fluctuations (ALFF). ALFF is expressed as *z*-values, relative to the global mean. Spearman correlation coefficient (*r*_s_) is overlaid on the figure

**Fig. 3:**
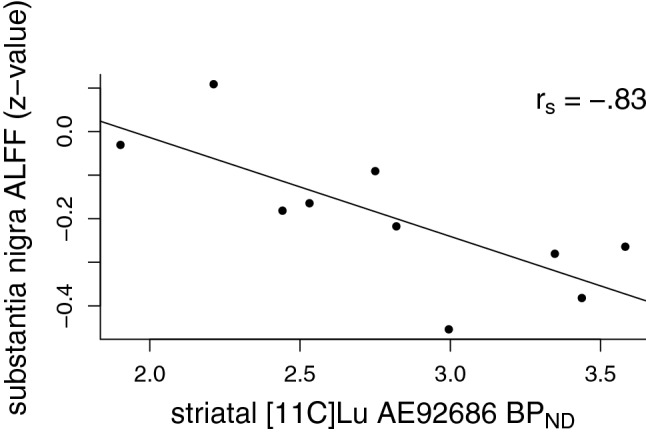
Scatter plot illustrating the correlation between mean striatal non-displaceable binding potential (BP_ND_) and mean substantia nigra amplitude of low-frequency fluctuations (ALFF). ALFF is expressed as *z*-values, relative to the global mean. Spearman correlation coefficient (*r*_s_) is overlaid on the figure

**Fig. 4:**
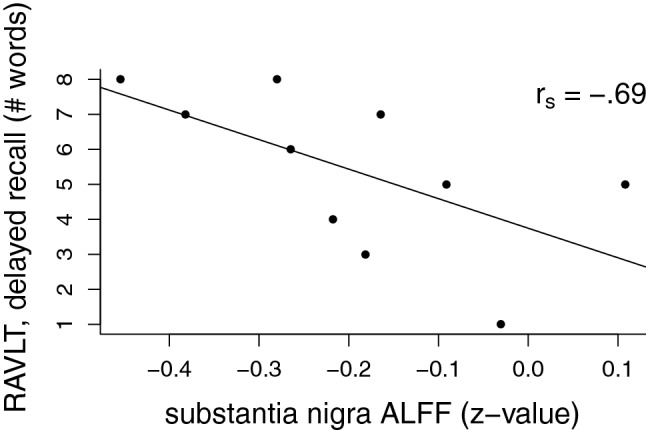
Scatter plot illustrating the correlation between mean substantia nigra amplitude of low-frequency fluctuations (ALFF) and the number of correctly recalled words during delayed recall in the Rey Auditory Verbal Learning Task (RAVLT). Spearman correlation coefficient (*r*_s_) is overlaid on the figure

Correlations between ALFF and striatal PDE10A are summarized in Table 1, and significant correlations are illustrated in Figs. 2, 3. Only putamen and substantia nigra ALFF showed a significant negative relationship with striatal PDE10A. No relationship was found in the caudate nucleus, nucleus accumbens or globus pallidus. Table 2 summarizes correlations between electrophysiological and behavioral measures and ALFF in the putamen and substantia nigra. There was a significant correlation between ALFF in the substantia nigra and delayed recall on RAVLT, with higher ALFF being associated with lower performance (see Fig. 4). No relationship between ALFF and MMN or TMT-B was found.

**Table 1:** Spearman correlations between mean striatal non-displaceable binding potential (BP_ND_), reflecting phosphodiesterase 10A (PDE10A) availability and the mean amplitude of low-frequency fluctuations (ALFF) within striatal and basal ganglia regions of interest

	ALFF, caudate nucleus	ALFF, putamen	ALFF, nucleus accumbens	ALFF, substantia nigra	ALFF, globus pallidus
PDE10A, striatum	0.35	− 0.72*	0.59	− 0.83**	− 0.37

*p<0.05; **p<0.01

**Table 2:** Spearman correlations between the mean amplitude of low-frequency fluctuations (ALFF) within the putamen and substantia nigra on the one hand, and the electrophysiological and behavioral measures on the other

	MMN	TMT-B	RAVLT
ALFF, putamen	0.18	0.12	− 0.40
ALFF, substantia nigra	0.14	0.24	− 0.69*

*MMN* mismatch negativity, *TMT*-*B* trail-making test B, *RAVLT* Rey Auditory Verbal Learning Test

*p<0.05

Median antipsychotic dosage was 13.75 mg per day olanzapine equivalents, with an interquartile range of 13.21 mg. Antipsychotic dosage (olanzapine equivalents) did correlate negatively with spontaneous activity in the caudate nucleus, rho=− 0.66, *p*=0.04, and the nucleus accumbens, rho=− 0.79, *p*=0.01. No significant relationship was observed in the putamen, rho=− 0.25, *p*=0.49, substantia nigra, rho=− 0.43, *p*=0.22, or globus pallidus, rho=− 0.27, *p*=0.45 (spearman correlations).

## Discussion

This study aimed to explore the relationship between striatal PDE10A levels and neural and behavioral function of striatal and downstream basal ganglia regions, in patients with schizophrenia.

Lower PDE10A levels in striatum were associated with higher ALFF in the putamen and substantia nigra. This is congruent with the fact that PDE10A inactivates cAMP and cGMP, thereby decreasing the excitability of MSNs [5]. The findings may therefore reflect an increased excitability in the putamen and substantia nigra as a consequence of the reduced PDE10A levels seen in our sample. This, in turn, is in line with earlier findings of a hyperactive putamen in schizophrenia, as observed in increased resting-state activity compared to controls [17–19].

Higher ALFF in the substantia nigra was related to lower verbal memory performance, in line with earlier findings of an association of episodic memory with both striatal dopamine receptors and substantia nigra activation [27, 28]. This may reflect a downstream effect of decreased PDE10A in the striatum, contributing to aberrant activity of the substantia nigra pars reticulata, in turn affecting episodic memory.

### Direct or indirect pathway?

The effects of PDE10A inhibition seem to predominate in the indirect pathway [10], exerting a dominant effect on D_2_-expressing neurons that project mainly via the globus pallidus and subthalamic nucleus [5, 43]. Our results demonstrate that increased putamen ALFF values are negatively correlated with striatal PDE10A. This is in agreement with both D_1_- and D_2_-mediated effects, as both the activated D_1_ and inhibited D_2_ pathways increase MSN excitability. However, we found that the ALFF of substantia nigra also shows a negative correlation with striatal PDE10 levels. This may imply that the downstream effects of PDE10A inhibition in the substantia nigra are mediated by the indirect pathway, in line with a dominant effect on D_2_-expressing MSNs. However, this simplified mechanistic explanation is challenged by the observation that D_1_ and D_2_ receptors are co-expressed by a significant percentage of medium spiny neurons as well, thus the interaction between these two receptors are likely more complex [44].

### Noise or signal?

One account of dopamine receptor-mediated modulation of the striatum is that it acts as a signal-to-noise regulator. Several studies demonstrated that increasing D_1_ activation would increase the signal-to-noise ratio of the D_1_ MSN, such that the neuron stops responding to weak inputs, but increases its response to strong inputs [45, 46]. Modeling studies suggested that D_1_ activation inhibits MSNs when already hyperpolarized, but excites MSNs when already depolarized [45]. Thus, the net effect of dopaminergic and PDE10 influences can be complex, depending on the state of the postsynaptic MSN neurons. Nevertheless, this implies that PDE10 may be important for augmenting the signal-to-noise ratio, and when decreased in schizophrenia, the MSNs are more depolarized, with even small inputs causing the cells to fire, making the system more susceptible to noise. In resting-state fMRI, increased activity has been found to co-occur with decreased local connectivity in schizophrenia [47], supporting the notion that increased activity may reflect increased noise rather than signal. This interpretation might explain the fact that higher activity within the substantia nigra was associated with lower verbal memory performance in this study.

### Consequence for PDE10A inhibitors

Intuitively, the finding of reduced striatal PDE10A in patients with schizophrenia would not predict a therapeutic effect of PDE10A inhibitors. However, PDE10A knockout mice do show impaired incentive salience [48], meaning that a chronic reduction of the enzyme may be detrimental as well. Both reduced and elevated cAMP levels, and consequently PDE10A, could impair signaling (e.g., [10]), implying a curvilinear relationship between PDE10A levels and function. It is worth noting that PDE10A inhibitors have shown limited efficacy in schizophrenia, suggesting that findings in preclinical models may not be directly translatable to human subjects (see https://clinicaltrials.gov/ct2/show/results/NCT01175135?sect=X01256#all). On the other hand, elevating cyclic nucleotides in symptomatic Huntington’s chorea animal models using PDE10A inhibitors restored basal ganglia function, despite a dramatic loss of the PDE10A enzyme [49]. They explain this counterintuitive finding with PDE10A decrease being compensatory to a decrease in cyclic nucleotides, which was restored with PDE10A inhibition. It is possible that the same mechanisms underlie our findings, which would reconcile a finding of decreased striatal PDE10A with a therapeutic potential of PDE10A inhibitors.

### Effect of medication or not?

Another possibility is that we observed an effect of medication. With our currently limited knowledge, the effects of chronic alterations in dopaminergic neurotransmission on PDE10A availability and binding potential are controversial. An animal study with rats found that repeated D-amphetamine treatments significantly increased PDE10A binding, which was not observed with selective D_1_ receptor blocking [50]. This might imply that chronic DA-related treatment modulates PDE10A levels in patients [51]. On the other hand, another rodent study found that a prior exposure to antipsychotic medication did not alter PDE10A levels [52]. In our study, while we did not find a relationship between striatal PDE10A levels and antipsychotic dosage, we did observe a negative relationship between ALFF and olanzapine equivalent dosage in the caudate and accumbens nuclei, but no relationship in the regions that actually showed a relationship with PDE10 level.

It seems that the dynamic pattern of dopaminergic input and the chronicity of the dopaminergic drug administration affects D_1_- or D_2_-dominated pathways differently [53]. PDE10A inhibition might exert D_2_-expressing neurons under basal conditions and, with increased dopaminergic transmission, the effects on D_1_-expressing neurons might predominate [48]. The effects of chronic D-amphetamine exposure in rats were found to be mediated by D_1_ over the D_2_ receptor pathway  [50]. Further studies are necessary to dissect the relation between chronic and state-dependent effects of antipsychotic medication and the altered PDE10A availability.

### Limitations

The sample in this study is quite small, limiting the generalizability of the findings. Due to the limited sample size, we have endeavored to keep the amount of significance tests to a minimum, only considering mean values from a priori regions of interest. Still, the reported correlations are evaluated at a non-corrected *p* value threshold and the results need to be considered with this in mind. Further, there was no control group with MRI or behavioral and electrophysiological data, making it impossible to say whether these measures deviated in patients. However, both episodic memory and striatal functioning have repeatedly been observed to deviate in patients with schizophrenia, something which is congruent with our findings.

Only two cognitive tests were included, of which one is sensitive to executive functioning. We cannot claim to have completely covered this complex construct with one test, and it is possible that the range of cognitive functions associated with a decrease in striatal PDE10A is wider than we show here.

The PET and MRI scanning sessions were separated in time, though held in close proximity and at a constant time of day between patients.

Due to limited spatial resolution and signal-to-noise ratio, conventional MR techniques, such as those used here, are not able to distinguish between different parts of the substantia nigra [54]. The substantia nigra pars reticulata is the larger part of the substantia nigra proper [55]. This makes it likely that the substantia nigra ALFF activity reflects mostly the downstream effect of decreased PDE10A in the striatum. However, if the substantia nigra ALFF value also reflects the substantia nigra pars compacta activity, it might imply that the increased dopaminergic effect on the striatum is associated with the decreased PDE10A levels. Given the opposite effects of dopamine on D_1_ and D_2_ medium spiny neuron excitability, this might indicate that with increased dopaminergic input there is decreased excitability in the D_2_ pathway [10]. This might lower PDE10 levels which in turn compensate by increasing (back) the excitability of MSNs. Thus, PDE10a downregulation may follow from overstimulation, which is in agreement with our finding where an increased putamen ALFF activity was correlated with reduced PDE10A levels. The limitations of this mechanistic explanation include known subpopulations of MSNs, which co-express both D_1_ and D_2_ receptors [44]. There are also inhibitory projections from the pars reticulata to the pars compacta [56], which further complicates the network.

## Conclusions

This study shows a negative relationship between striatal PDE10A and basal ganglia functioning, both at the neural and behavioral level, in patients with schizophrenia who show reduced PDE10A levels. The findings are in line with a role of PDE10A in striatal neuron functioning and may be informative in the development of new treatments.
